# Identification of Wild-Type *CYP321A2* and Comparison of Allelochemical-Induced Expression Profiles of *CYP321A2* with Its Paralog *CYP321A1* in *Helicoverpa zea*

**DOI:** 10.3390/insects12010075

**Published:** 2021-01-15

**Authors:** Shengyun Li, Song Chen, Xingcheng Xie, Shuanglin Dong, Xianchun Li

**Affiliations:** 1 Key Laboratory of Integrated Management of Crop Diseases and Pests (Ministry of Education), College of Plant Protection, Nanjing Agricultural University, Nanjing 210095, China; 2015202026@njau.edu.cn; 2 Department of Entomology, University of Arizona, Tucson, AZ 85721, USA; songchen75@gmail.com (S.C.); xingchengxie@email.arizona.edu (X.X.); 3 School of Agricultural Sciences, Zhengzhou University, Zhengzhou 450001, China; 4 State Key Laboratory for Biology of Plant Diseases and Insect Pests, Institute of Plant Protection, Chinese Academy of Agricultural Sciences, Beijing 100193, China

**Keywords:** *Helicoverpa zea*, *CYP321A2*, resistance, duplicate genes, allelochemical-induced expression

## Abstract

**Simple Summary:**

Plant toxin- and insecticide-detoxifying genes known as P450s are often involved in insect resistance to xenobiotics. For polyphagous generalists, investigation of ecologically significant P450s and their induced expression profiles by allelochemicals is of particular importance to understand the roles of these genes in detoxification of allelochemicals and insecticides, and the adaptation of generalists to their chemical environment. Previous studies identified an allelochemical-inducible P450 gene *CYP321A1* in polyphagous *Helicoverpa zea*, which is associated with detoxification of its inducers including plant allelochemicals and insecticides. Our study represents the identification, features of *CYP321A2* (a duplicated paralog of *CYP321A1*), as well as the responses of *CYP321A* paralogs to allelochemicals and plant signal molecules in *H. zea*. Moreover, xanthotoxin- and flavone-responsive regulatory elements of *CYP321A1* were also detected in the promoter region of *CYP321A2*. Our results enrich the P450 inventory by identifying an allelochemical broadly induced *CYP321A2* in *H. zea*. Our data also suggest that the *CYP321A2*/*CYP321A1* paralogs are a pair of duplicated genes of multigene families and *CYP321A2* has the potential to detoxify plant allelochemicals and adapt to its chemical environment.

**Abstract:**

One possible way to overcome the diversity of toxic plant allelochemicals idiosyncratically distributed among potential host plants is to have more counterdefense genes via gene duplication or fewer gene losses. Cytochrome P450 is the most important gene family responsible for detoxification of the diversity of plant allelochemicals. We have recently reported the identification and cloning of the transposon (*HzSINE1*)-disrupted non-functional *CYP321A2*, a duplicated paralog of the xenobiotic-metabolizing P450 *CYP321A1* from a laboratory colony of *Helicoverpa zea*. Here we report the identification of the wild-type intact allele of *CYP321A2* from another *H. zea* colony. This *CYP321A2* allele encodes a deduced protein of 498 amino acids and has the P450 signature motifs. Quantitative RT-PCR experiments showed that this *CYP321A2* allele was highly expressed in midgut and fat body and achieved the highest expression level in the developmental stage of 5th and 3rd instar larvae. *CYP321A2* and *CYP321A1* were constitutively expressed in low levels but can be differentially and significantly induced by a range of the plant allelochemicals and plant signal molecules, among which xanthotoxin, flavone, and coumarin were the most prominent inducers of *CYP321A2* both in midgut and fat body, whereas flavone, coumarin, and indole-3-carbinol were the prominent inducers of *CYP321A1* in midgut and fat body. Moreover, xanthotoxin- and flavone-responsive regulatory elements of *CYP321A1* were also detected in the promoter region of *CYP321A2*. Our results enrich the P450 inventory by identifying an allelochemical broadly induced *CYP321A2*, a paralog of *CYP321A1* in *H. zea*. Our data also suggest that the *CYP321A2*/*CYP321A1* paralogs are a pair of duplicated genes of multigene families and *CYP321A2* could potentially be involved in the detoxification of plant allelochemicals and adaptation of *H. zea* to its chemical environment.

## 1. Introduction

Herbivores display considerably more variability in the breadth of their host plant range [1]. Oligophagous specialists are highly specialized on a relatively narrow range of host plants (three or fewer plant families) and encounter a narrow range of allelochemicals [2,3], while polyphagous generalists are generalized on a wide range of plant families thereby encounter a diverse array of biosynthetically distinct allelochemicals. Although polyphagous herbivores may have relative fewer limitations in terms of food availability, the toxicological challenge of generalized feeding on host plants is considerable in that they have more possibilities to encounter various plant defense compounds (allelochemicals) [4]. How generalists cope with the large variety of toxins present in their host plants remains largely unknown.

Insect herbivores rely to some extent on counterdefense genes for detoxification of plant allelochemicals. In order to adapt to a broad and unpredictable array of plant defenses, counterdefense genes from generalists have multiple functions and have a much wider substrate spectrum than those from specialists. Among the counterdefense genes cytochrome P450 monooxygenases (P450s) play the most dominant role [5]. P450s are a superfamily of heme-binding enzymes that play a major role in metabolizing endogenous substrates (steroid hormones, lipids, etc.) as well as xenobiotics (plant allelochemicals, insecticides, etc.) [6]. Plants employ P450s to synthesize toxic defensive compounds known as allelochemicals to defend themselves against herbivorous insects, whereas insect herbivores use P450s to detoxify plant allelochemicals they encounter in their host plants [7,8]. The efficacy of detoxification depends on the levels of P450 transcripts expressed after exposure, the range of chemicals capable of inducing expression, and the turnover rates of individual P450 proteins involved in detoxification, among which the capability of being induced by foreign chemicals or “xenobiotics” is one remarkable feature of P450 genes [6,9]. For herbivorous insects, investigating ecologically significant P450s and their induced expression profiles by allelochemicals and plant signal molecules is of particular importance to understand the roles of these genes in detoxification of allelochemicals and insecticides, and the adaptation of insects to the chemical environment.

Gene duplication and gene conversion have long been implicated in the evolution of multigene families [10,11,12,13]. The former produces duplicated copies whereas the latter reduces the rate of pseudogene formation and allows duplicated copies to acquire selectable differences in protein sequences and regulatory sequences [14]. The P450 gene superfamily is a family replete with duplication, conversion, and subsequent divergence events [9]. In the corn earworm, *Helicovoerpa zea*, a polyphagous noctuidae of economic importance, only a few counterdefense genes have been well characterized. Perhaps the best characterized are allelochemical-metabolizing P450s in the *CYP6B* subfamily isolated from larvae of *H. zea*. Gene duplication and conversion events have played a role in the evolution of this *H. zea CYP6B* subfamily. Four *CYP6B* genes, *CYP6B8*, *CYP6B9*, *CYP6B27* and *CYP6B28*, exist in the *H. zea* genome as two pairs of paralogs that evolved from gene duplication and 5’-polar gene conversion events [9]. Among the P450 transcripts examined, *CYP6B8* and *CYP6B28*, with extremely high amino acid identity (99.8%), is a pair of highly conserved paralogs mainly expressed in midgut and fat body [9,15]. The relatively divergent *CYP6B9* and *CYP6B27* transcripts (97.4% amino acid identity) are derived from another pair of paralogous P450 genes (87% amino acid identity with *CYP6B8*), whose expression is restricted to midgut [9,15]. The four *CYP6B* genes are all expressed constitutively in midgut of all larval instars and differentially induced in response to a number of xenobiotics. All of them were strongly induced by plant allelochemicals including indole-3-carbinol and chlorogenic acid [9], and plant defense signaling compounds including jasmonate and salicylate [16], strongly implicating all four of these *CYP6B* genes are involved in metabolism of host plant allelochemicals. Their common origins and high amino acid sequence identities indicate that they are xenobiotic-metabolizing P450s since CYP6B8 protein has been proven to metabolize a wide range of plant allelochemicals (xanthotoxin, quercetin, flavone, chlorogenic acid, indole-3-carbinol and rutin) and synthetic insecticides (diazinon, cypermethrin and aldrin) [4]. The high degree of conservation that the four *H. zea* P450 genes share in their coding sequences argues for a high degree of similarity in their catalytic properties. Divergence in the expression patterns of these genes has the potential to assist in the acquisition of novel function for these duplicated copies. The presence of such multiple, closely related P450 genes within a genome appears to be widespread among insects [17,18].

In the same species, another well-identified P450 is *CYP321A1*, a P450 which is highly induced in larval midgut in response to plant allelochemicals flavone and xanthotoxin but not insecticide cypermethrin [19,20,21,22]. *CYP321A1* is also involved in the detoxification of plant allelochemicals (xanthotoxin, angelicin and *α*-naphthoflavone), insecticide (*α*-cypermethrin, aldrin and diazinon) and aflatoxin B1 by baculovirus-mediated functional expression analysis [19,23,24]. A duplicated copy of this gene, termed *CYP321A2*, was identified and characterized here. In this study, we identified the wild-type intact *CYP321A2* in the tested lab colony and compared the coding and 5′-flanking sequences between two *CYP321A* paralogs. Tissue- and developmental stage-specific expression of *CYP321A2* and the allelochemical- and plant signal molecule-induced expression profiles of both *CYP321A2* and *CYP321A1* were investigated by quantitative RT-PCR. The results obtained suggest that *CYP321A2* in *H. zea* is a functional intact P450 and has the potential to deal with the diversity of plant allelochemicals.

## 2. Materials and Methods

### 2.1. Insects and Plant Xenobiotics Induction

**Figure 1:**
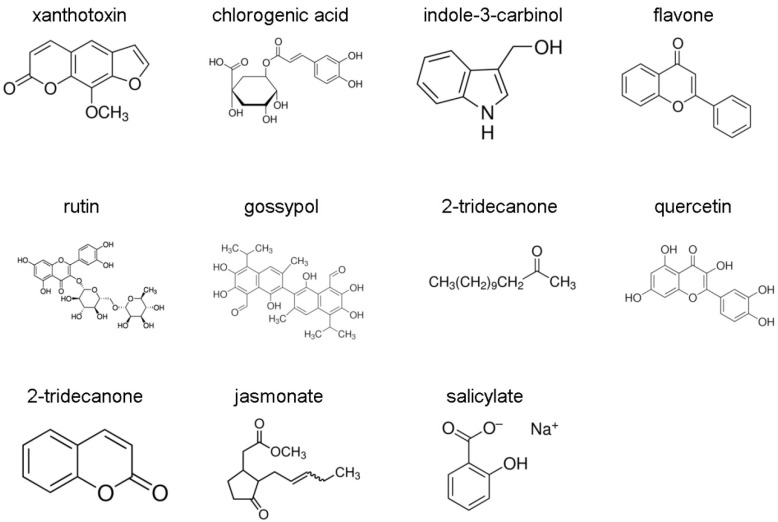
Allelochemicals and plant signal molecules tested in the inducible expression of *CYP321A2* and *CYP321A1* in *H. zea*. Molecular structures of allelochemicals and plant signal molecules were from Sigma-Aldrich website (https://www.sigmaaldrich.com).

A laboratory colony of *H. zea*, generously provided by Dr. May R. Berenbaum (Department of Entomology, University of Illinois at Urbana-Champaign), was maintained in an insectary kept at 28 °C with a photoperiod of 16 h light: 8 h dark on a semi-synthetic control diet containing wheat germ [25]. Induction treatments were performed as described by Li et al. [9]. The analytical grade plant allelochemicals, xanthotoxin, chlorogenic acid, indole-3-carbinol, flavone, rutin, gossypol, 2-tridecanone, quercetin, coumarin and plant signal molecules jasmonate and salicylate, used in this study, were obtained from Sigma (Sigma-Aldrich, St. Louis, MO, USA) (Figure 1). In brief, 30 newly molted 5th instar larvae were allowed to feed on control diets or control diets containing 0.1% plant xenobiotics for 48 h. Three independent biological replicates of the control diet or each plant xenobiotic treated diet were prepared for subsequent RNA extraction. Midguts and fat bodies were then dissected out, flash-frozen in liquid nitrogen, and stored at −80 °C for subsequent RNA extraction.

### 2.2. DNA Extraction and Cloning of Genomic Sequences

Genomic DNA was isolated from the 5th instar larvae using the procedure described by Sambrook and Russell [26]. The 5′-flanking promoter sequence of *CYP321A1* was obtained by the Universal Genome Walker kit (Clontech, Mountain View, CA, USA) according to the manufacturer’s manual. In brief, genomic DNA was digested by several restriction enzymes and then ligated to the genome walking adaptor. The resulting DNA fragments were used as templates to PCR-amplify the 5′-flanking sequence of *CYP321A1* using the two general forward primers adaptor primer 1 (AP1) and nested adaptor primer 2 (AP2) complementary to the adaptor sequences and the two corresponding gene-specific reverse primers Hz321A1GSP1 (ACCGATCAGGTACCACGTTAGTAAGAG) and Hz321A1GSP2 (ATCTCGAGCCTAATAAAATCAGTGGTAGTTGTAAC). The reaction mixture contained 5 μL 10× reaction buffer, 5 μL MgCl_2_ (25 mM), 1 μL dNTPs (10 mM), 2 μL gene-specific primer (10 μM), 2 μL AP1 (10 μM) (AP2 for the secondary PCR), 1 μL genomic DNA sample (1 μL of 50× diluted primary PCR product as template in the secondary PCR), 0.5 μL mixture (10:1) of Taq DNA polymerase (Thermo Scientific, Waltham, MA, USA) and Pfu DNA polymerase (Stratagene, La Jolla, CA, USA) and the final volume was adjusted with sterile water to 50 μL. The nested PCR reactions began with the primary PCR consisting of 25 cycles of 94 °C denaturation for 2 min, 68 °C annealing/extension for 4 min, followed by the secondary PCR consisting of 35 cycles of 94 °C denaturation for 2 min and 68 °C annealing/extension for 4 min. PCR products were run on a 1.0% agarose gel in 1 × TAE buffer. The longest band was eluted from the gel using the QIAquick Gel Extraction Kit (Qiagen, Valencia, CA, USA) and then directly cloned into the pGEM^®^-T Easy Vector (Promega, Madison, WI, USA). One positive clone was sequenced on Applied Biosystems 3730 DNA Analyzer (Thermo Scientific, Waltham, MA, USA) twice in both directions using M13 forward and M13 reverse primers as well as internal primers designed on the basis of the determined sequences at the Genomic Analysis & Technology Core Facility of the University of Arizona.

### 2.3. RNA Extraction and First Strand cDNA Synthesis

Total RNA was isolated from different tissues (integument, midgut, fat body and ovary) of the 5th instar larvae, larvae of different developmental stages (pupae, neonates, 3rd larvae, 5th larvae and adults), and thirty caterpillar midguts/fat bodies dissected from larvae fed on control diet or plant xenobiotic-treated diet using the guanidinium-HCl procedure [27]. Three independent biological replicates were prepared for expression analysis. DNase I (RNase-free) (New England Biolabs, Ipswich, MA, USA) was used to eliminate the potential genomic DNA contamination in the RNA samples. Two micrograms of the isolated total RNA in each sample was used as template for first strand cDNA synthesis with oligo-dT_18_ primer and M-MLV Reverse Transcriptase (Promega, Madison, MI, USA) following the manufacturer’s instructions. The cDNA synthesized here was ready for the downstream quantitative RT-PCR.

### 2.4. Identification of CYP321A2 Transcript by 5′- and 3′- RACE

For identification of the intact *CYP321A2* transcript in the tested laboratory colony of *H. zea*, 5′- and 3′-rapid amplification of cDNA ends (RACE) were conducted using the SMART™ RACE cDNA Amplification Kit (Clontech, Mountain View, CA, USA) as described by the manufacturer. Briefly, the first strand 5′-RACE-Ready cDNA and 3′-RACE-Ready cDNA were synthesized with BD PowerScript Reverse Transcriptase using one microgram of the control sample as templates. The synthesized first strand 5′-RACE-Ready cDNA was employed as template to PCR-amplify the 5′ end of *CYP321A2* cDNA using the Universal Primer A Mix (UPM) and the Nested Universal Primer A (NUP) complementary to the adapter sequences and the two corresponding *CYP321A2*-specific reverse primers N321GSP3 (5′- ACCATT TTTTTGAAGGCTGA-3′) and N321GSP4 (5′-GAAGAATGCCTG TGCC GCTA-3′). Likewise, the synthesized 3′-RACE-Ready cDNA was employed as template to PCR-amplify the 3′ end of *CYP321A2* cDNA using UPM and NUP and two *CYP321A2*-specific forward primers N321GSP2 (5′-CGATGATGATGGACCCCGA-3′) and 321A1DL7SF1 (5′-GAGGAACGATTTCGCTGATT-3′). For both 5′- and 3′-RACE, the nested PCR reactions processed with the primary PCR consisting of 20 cycles of 94 °C denaturation for 30 s, 60 °C annealing for 30 s, and 72 °C extension for 2 min, followed by the secondary PCR consisting of 35 cycles of 94 °C denaturation for 30 s, 60 °C annealing for 30 s and 72 °C extension for 2 min. The 5′- and 3′-RACE PCR products were electrophoresed on a 1.0% agarose gel in 1× TAE buffer. The resultant bands were individually eluted from the gel using the QIAquick Gel Extraction Kit (Qiagen, Valencia, CA, USA), directly cloned into the pGEM^®^-T easy vector (Promega, Madison, MI, USA), and then sequenced at the Genomic Analysis & Technology Core Facility of the University of Arizona.

### 2.5. Analysis of CYP321A2 Expression Level in Different Tissues and Developmental Stages

Quantitative RT-PCR (RT-qPCR) assays were used to determine the *CYP321A2* expression levels in different tissues of 5th instar larvae and developmental stages of *H. zea.* A pair of specific primers yielding short PCR product was designed using online software Primer3 (https://bioinfo.ut.ee/primer3-0.4.0/) for *CYP321A2* and a standard housekeeping gene *EF-1α*. The forward primer R321A2F (5′-AGTCTTGCGCCAAGTTTGAT-3′) and reverse primer R321A2R (5′-CACCCGCTGAGAAGAAGAAG-3′) were used to amplify *CYP321A2*. The forward primer REF-1F (5′-GCCTGGTACCATTGTCGTCT-3′) and reverse primer REF-1R (5′-GTAACCACGACGCAACTCCT-3′) were used to amplify *EF-1α*. Validation experiments showed that the *CYP321A2* and *EF-1α* primer sets had an approximately equal efficiency of amplification (data not shown). PCR reactions were performed in ABI 7300 Real-Time PCR System (Applied Biosystems, Foster City, CA, USA) by using qPCR MasterMix Plus for SYBR Green I kit (Eurogentce, Fremont, CA, USA). The 25 μL reaction mixture contained 12.5 μL 2× reaction buffer, 1 μL forward primer, 1 μL reverse primer, 1 μL 20× diluted cDNA sample, and final volume was adjusted with sterile water. The thermal cycling conditions were first 50 °C for 2 min and 95 °C for 10 min, then 45 cycles of 95 °C denaturation for 15 s and 60 °C annealing/extension for 1 min, and a final standard dissociation stage of 95 °C for 15 s, 60 °C for 30 s, and 95 °C for 15 s. The final dissociation stage was included to yield dissociation curves for verifying the specificity of the amplification products. Reporter fluorescence signal data were collected once per each PCR circle during the 60 °C annealing/extension step. Each sample was repeated three times. Relative *CYP321A2* gene expression, normalized to the endogenous standard housekeeping gene *EF-1α* and relative to a calibrator (integument for the tissue expression, neonates for the developmental stage expression), was calculated by comparative Ct (threshold cycle) method (ΔΔCt) by the formula 2^−(ΔΔCt)^, where ΔΔCt = (Ct*_CYP321A2_* − Ct*_EF-1α_*) _test sample_ − (Ct*_CYP321A2_* − Ct*_EF-1α_*) _calibrator sample_ [28].

### 2.6. Allelochemical and Plant Signal Molecule Induced Expression of CYP321A2 and CYP321A1 in Midgut and Fat Body

Inducible expression of *CYP321A2* and *CYP321A1* by different allelochemicals and plant signal molecules in *H. zea* midgut and fat body was analyzed by RT-qPCR. The primers used for *CYP321A2* and *EF-1α* are described as above. The forward primer R321A1F (5′-CAAAGCGTATAGAAATGAGCCGG-3′) and reverse primer R321A1R (5′-TTTCGCATTAACTTCCACTTGGG-3′) were used to amplify *CYP321A1*. Validation experiments showed that the *CYP321A2*, *CYP321A1* and *EF-1α* primer sets had an approximately equal efficiency of amplification (data not shown). RT-qPCR method was described as above. Each sample was repeated three times. Dissociation curves were applied to verify the specificity of amplification products after PCR. Relative *CYP321A2* and *CYP321A1* expression in midgut and fat body, normalized to the endogenous standard housekeeping gene *EF-1α* and relative to a calibrator (untreated group), was calculated by comparative Ct (threshold cycle) method (ΔΔCt) by the formula 2^−(ΔΔCt)^, where ΔΔCt = (Ct*_CYP321A2/CYP321A1_* − Ct*_EF-1α_*) _plant xenobiotic treated group_ − (Ct*_CYP321A2/CYP321A1_* − Ct*_EF-1α_*) _untreated group_ [28].

### 2.7. Sequence Analysis

The *CYP321A2* sequence obtained by RACE method was compared with *CYP321A1* sequence (GenBank accession no. AY113689.1) to check their identity. Nucleotide sequence alignment was conducted to identify the conserved elements and divergence in 5′ flanking region of the *CYP321A2* and *CYP321A1* (GenBank accession no. DQ788841). Amino acid sequence alignment of *CYP321A2* and *CYP321A1* (GenBank accession no. AAM54724.1) was conducted to locate the six substrate recognition sites (SRSs) and the conserved motifs of P450 using DNAMAN version v6 software.

### 2.8. Statistical Analysis

A two-tailed Student’s *t*-test was used to compare *CYP321A2/CYP321A1* mRNA expression levels between xenobiotic-treated groups and the untreated control group. Significant differences among expression levels of *CYP321A2* in different tissues and developmental stages were determined by one-way analysis of variance (ANOVA) followed by Tukey’s HSD tests for multiple comparisons.

## 3. Results

### 3.1. Identification of the Wild-Type Intact CYP321A2

**Figure 2:**
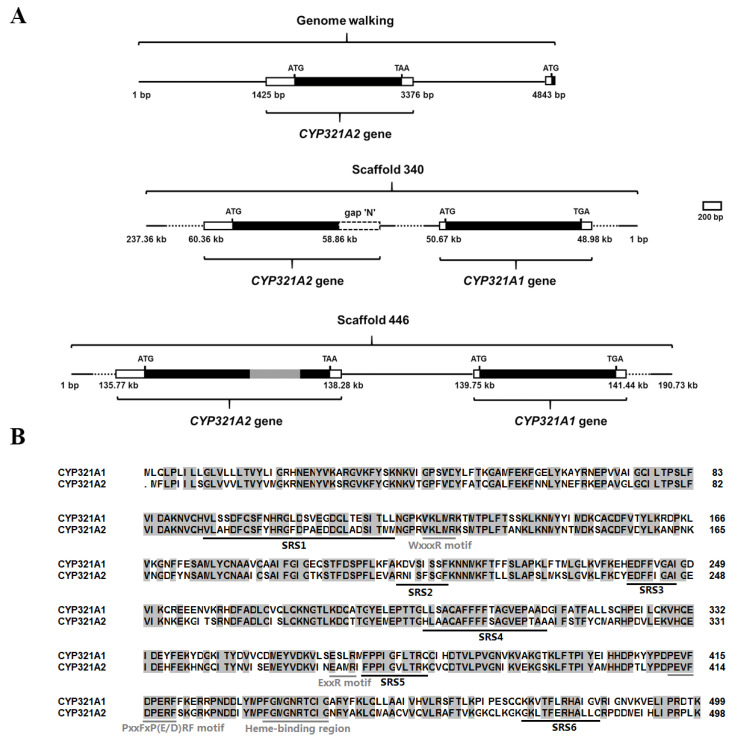
Relative positions of *CYP321A2* with *CYP321A1* in genome walking sequence, *H. zea* genome scaffold 340 and scaffold 446 (**A**) and their amino acid sequence alignment (**B**). (**A**). Numbers below the maps indicate the relative positions of each gene in the scaffold. Black, white and grey boxes represent the coding region, UTR region and transposon, respectively. Dotted white box represents the region not found in the scaffold because of the presence of gap “N”. The start codon ATG and stop codon TGA/TAA were also indicated. (**B**). Black solid underlines indicate putative substrate recognition sites (SRSs) based on analogy from a comparative analysis of *CYP321A1* and *CYP6B8* [23]. Gray solid underlines indicate consensus sequences of the conserved P450 motifs: helix-C (WxxxR), helix-K (ExxR), PxxFxP(E/D)RF motif and the heme-binding region (FxxGxRxCxG), where x is any amino acid [29,30].

**Figure 3:**
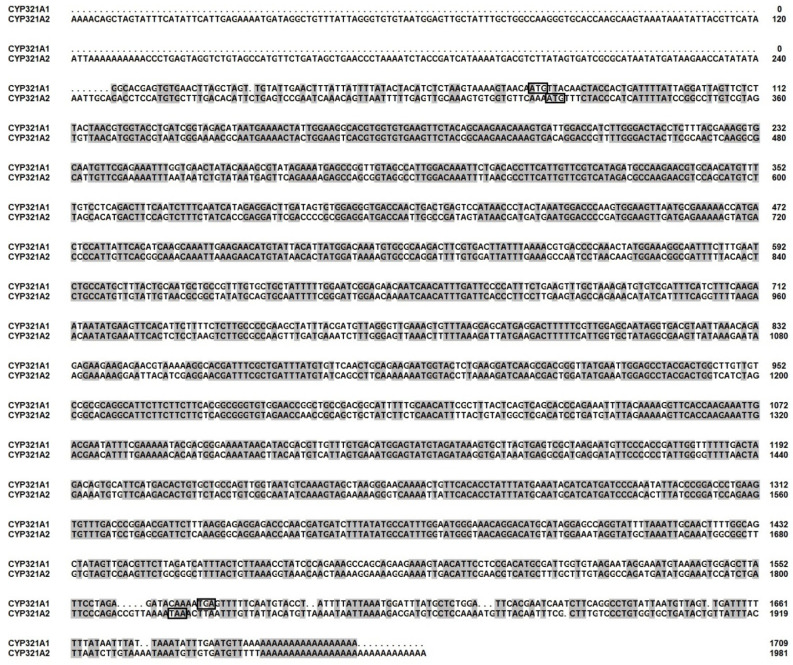
Nucleotide alignment of wild-type *CYP321A2* with its paralog *CYP321A1*. All the consensus nucleotides are shaded in gray. The ATG start codon and the TGA or TAA stop codon are black boxed.

At the time of experiments the 5′-flanking promoter sequence of the *CYP321A1* was not availabale (i.e., only *H. zea* scaffold 340 (Clarke et al., April 2017) was available) and hence 5′ RACE and genome walking experiments were performed to determine the sequence of this region. Sequence analysis showed that this genomic walking product not only contained the 5′ promoter sequence of *CYP321A1*, but also contained another open reading frame encoding a deduced protein of 498 amino acids located 1467-bp upstream of *CYP321A1* (Figure 2A). To study the transcript of this gene in the tested laboratory colony, the rapid amplification of cDNA ends (RACE) approach was employed. A transcript of 1982-bp was characterized. The transcript contains a 323-bp 5′ UTR, a 162-bp 3′ UTR and an open reading frame (1497-bp) encoding a deduced protein of 498 amino acids (GenBank accession no. MN402503) (Figure 2B and Figure 3). The predicted molecular mass and pI of its deduced protein are 56.68 kDa and 8.90, respectively (https://web.expasy.org). BLAST search showed that this is a P450 that shares the highest amino acid identity (67.1%) with *CYP321A1* and thus is designated *CYP321A2* (D. Nelson, personal communication). The *CYP321A2* has conserved C-helix WxxxR motif, the ExxR motif in the K-helix, the conserved motif PxxFxP(E/D)RF which is located after the K-helix, and the canonical P450 heme-binding region FxxGxRxCxG [29,30] (Figure 2B), suggesting that it is a wild-type intact P450 in *H. zea*.

Consistent with our genome walking data, the two genes are indeed tandemly arranged in a head-to-tail orientation in scaffold 340 from the *H. zea* genome assembly (Clarke et al., April 2017. GenBank accession no. KZ118142.1; https://www.ncbi.nlm.nih.gov/assembly/GCA_002150865.1) and scaffold 446 from another *H. zea* genomic sequence assembly (Perera et al., June 2020. GenBank accession no. MT702953.1; https://www.ncbi.nlm.nih.gov/nuccore/MT702953.1), with *CYP321A2* located upstream of *CYP321A1* (Figure 2A). Since the two scaffolds were from different populations of *H. zea*, they represent two allelic variants of the same *loci* in the *H. zea* genome. Scaffold 340 contains an intact wild-type *CYP321A2* allele, whereas scaffold 446 has a transposon-inserted *CYP321A2* allele (Figure 2A).

### 3.2. Tissue-Specific and Developmental Expressions of CYP321A2

**Figure 4:**
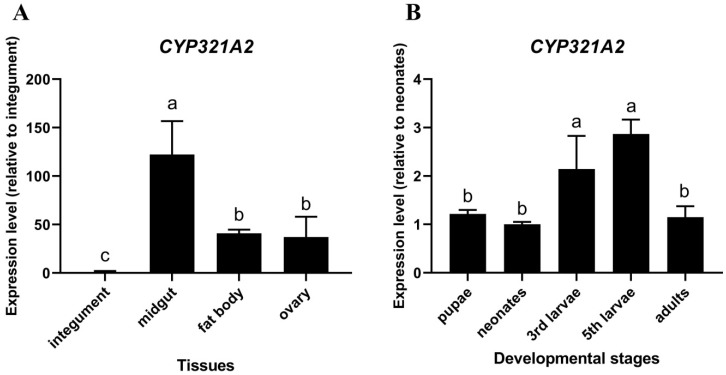
Expression patterns of *H. zea CYP321A2* gene in different tissues of the fifth-instar larvae and in the different developmental stages as evaluated by RT-qPCR. (**A**) Expression levels were calculated relative to integument in midgut, fat body and ovary. (**B**) Expression levels were calculated relative to neonates in pupae, 3rd larvae, 5th larvae and adults. The error bars indicate 95% confidence limits of the means (n = 3). Values sharing the same letter are not significantly different at *p* < 0.05 (Tukey’s HSD tests).

Because tissue- and developmental stage-specific expression patterns of P450 genes in insects may be related to their roles in the particular tissues and developmental stages, the expression levels of *CYP321A2* were determined in different tissues of the 5th instar larvae and developmental stages of *H. zea* by using the RT-qPCR approach. The results showed that the *CYP321A2* expression level in midgut was the highest (122.2-fold comparing to the integument) among the four tissues tested here, followed by fat body and ovary (40.9-fold and 37.1-fold comparing to the integument, respectively) (Figure 4A). For the *CYP321A2* expression in different developmental stages, 5th and 3rd instar larvae have the highest *CYP321A2* expression level (2.9-fold and 2.1-fold comparing to the neonates, respectively) (Figure 4B). However, in pupae and adults, the expression levels of *CYP321A2* were only slightly higher than the level in the neonates (1.2-fold and 1.1-fold, respectively) (Figure 4B).

### 3.3. Allelochemical and Plant Signal Xenobiotic Induced Expressions of CYP321A2 and CYP321A1

*H. zea* is a polyphagous insect that encounters in its diet several plant toxic compounds. As P450s are known to be expressed at low levels and to be inducible when insects have to deal with toxic compounds, *CYP321A2* and *CYP321A1* induction profiles in midgut and fat body were measured in larvae exposed to plant allelochemicals (xanthotoxin, chlorogenic acid, indole 3-carbinol, flavone, rutin, gossypol, 2-tridecanone, quercetin and coumarin) and plant signal molecules (jasmonate and salicylate) (Figure 1). RNA samples from 5th instar larvae fed for 48 h on control diet or diets supplemented with 0.1% allelochemicals or plant signal molecules were analyzed by RT-qPCR. Almost all the plant compounds up-regulated the expressions of *CYP321A2* and *CYP321A1* in varying degrees in midgut and fat body (Table 1). Out of 11 inducers analyzed, xanthotoxin, chlorogenic acid, indole-3-carbinol, flavone, rutin, quercetin and coumarin were the common inducers for both genes in either midgut or fat body. Salicylate was also the effective inducer of *CYP321A2*, whereas 2-tridecanone and jasmonate only effectively induced the expression of *CYP321A1*. Flavone is the most potent inducer for both *CYP321A2* and *CYP321A1,* causing the highest upregulations (850.76-fold for *CYP321A2* in midgut and 306.82-fold for *CYP321A1* in midgut), followed by coumarin (61.18-fold for *CYP321A2* in midgut and 102.36-fold for *CYP321A1* in midgut). Compared to the induction of *CYP321A1*, xanthotoxin resulted in a much higher induction of *CYP321A2*. By contrast, the expression level of *CYP321A1* was notably higher than that of *CYP321A2* in response to indole-3-carbinol and quercetin. Chlorogenic acid, rutin and coumarin have a similar induction effect on the expression of *CYP321A2* and *CYP321A1* in midgut and fat body. 2-tridecanone, jasmonate and salicylate in fat body, jasmonate in midgut slightly repressed (not significantly) the expressions of *CYP321A2* and *CYP321A1*, respectively. Gossypol had no influence on the expressions of both genes in midgut or fat body (Table 1).

### 3.4. Sequence Comparison in the 5′ Flanking Regions of CYP321A2 and CYP321A1

**Figure 5:**
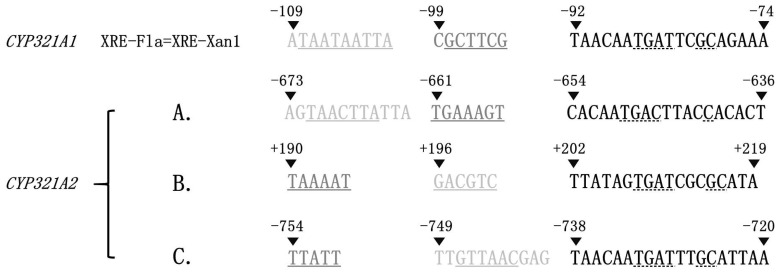
Sequence alignment between the essential flavone- and xanthotoxin-responsible element XRE-Fla in *CYP321A1* and its analogs in *CYP321A2*. XRE-Fla in *CYP321A1* is composed of three motifs: the AT-only TAAT inverted repeat (Motif 1, light grey), the GC-rich GCT mirror repeat (Motif 2, dark grey) and the ARE-like element (Motif 3, black) [31,32]. Three XRE-Fla-like elements are found in the 5′ UTR and promoter region of *CYP321A2* and shown below the *CYP321A1* XRE-Fla with the same colors to indicate their three motifs. The patterns in inverted repeat and mirror repeat are in solid underlines. The conserved nucleotides in ARE-like elements are in dashed underlines. The nucleotides are numbered relative to the transcription start site (TSS, +1), with sequence upstream of it preceded by “−”, and downstream of it preceded by “+”.

The greater flavone and xanthotoxin inducibility of *CYP321A2* than *CYP321A1* (Table 1) suggests that one or more copies of XRE-Fla (also known as XRE-Xan1), the essential *cis*-acting element mediating the induction of the allelochemical-metabolizing *CYP321A1* by flavone and xanthotoxin [31,32], may be present in the 5′ flanking genomic sequence of *CYP321A2*. XRE-Fla in *CYP321A1* is composed of the AT-only TAAT inverted repeat (Motif 1, light grey), the GC-rich GCT mirror repeat (Motif 2, dark grey) and the ARE-like element (Motif 3, black) (Figure 5) and its function is determined by the sequence of Motif 3 and the repeat types of Motif 1 and 2, rather than their sequences [31,32]. Manual search of the 5′ flanking genomic sequence of *CYP321A2* with the above functional determinants of XRE-Fla found three XRE-Fla-like elements (A, B and C in Figure 5). Like *CYP321A1* XRE-Fla, all the three *CYP321A2* XRE-Fla analogs contain an ARE-like element, an inverted repeat and a mirror repeat, but the sequences of their inverted and mirror repeats are different from each other and from those of *CYP321A1* XRE-Fla (Figure 5). The three motifs of the A analog are arranged in the same order (inverted repeat, followed by mirror repeat and then ARE-like element) as in the *CYP321A1* XRE-Fla, whereas the inverted repeat motif trades position with the mirror repeat motif in the B and C analogs.

## 4. Discussion

In order to survive, herbivorous insects must deal with both naturally occurring plant toxins and synthetic insecticides in their diets. Relative to specialist herbivores, generalist herbivores face a large diversity and unpredictability of toxic plant allelochemicals [33]. One possible way for generalists to overcome the diversity of toxic plant allelochemicals distributed among potential host plants is to have more counterdefense genes via gene duplication or fewer gene losses for detoxification of plant allelochemicals [4]. In this study, we have enlarged the known P450 inventory in *H. zea* with one new cytochrome P450 monooxygenase: the wild-type intact *CYP321A2*, a paralog of previously identified *CYP321A1*, which is involved in the metabolism of plant allelochemicals and insecticides in *H. zea* [19,23,24]. *CYP321A2* identified here in the tested laboratory strain located 1467-bp upstream of *CYP321A1* and encoded a deduced protein of 498 amino acid residues, which is only one amino acid shorter than *CYP321A1* (499 amino acid residues) (Figure 2B). It has 67.1% amino acid identity with *CYP321A1* and shares several identical P450 conserved motifs with *CYP321A1* (Figure 2B). The two genes are tandemly arranged in head-to-tail orientation in *H. zea* genome scaffolds (Figure 2A). These data indicate that the *CYP321A2* gene is a wild-type intact P450 and apparently a duplicated paralog of *CYP321A1* in this tested colony of *H. zea*. The insect midgut is a particularly rich source of P450 activity [34]. RT-qPCR analyses have shown that *CYP321A2* gene is expressed constitutively in midgut, 3rd and 5th instar larvae (Figure 4). The role of midgut in detoxification processes is well-known and P450s identified in it may be important players in these processes. It was speculated that the highest expression of *CYP321A2* in larvae stage (3rd and 5th instar) might be due to the adaptive regulation of the insect to metabolize xenobiotics upon exposures. Because of dramatically increased food uptake in 3rd and 5th instar stage especially in 5th instar nymphs of the *H. zea*, up-regulation of *CYP321A2* gene may help metabolize toxic plant secondary metabolites that they encounter in their diet.

The capacity to induce expressions of their counterdefense genes in response to a wide range of structurally distinct allelochemicals is essential for generalist herbivores to overcome the uncertainty and unpredictability of plant defenses [32]. Previously, there are two sets of paralogous cytochrome P450 genes *CYP6B8*/*CYP6B28* and *CYP6B9*/*CYP6B27* evolved from gene duplication and 5′-polar gene conversion events were identified in *H. zea* and these four *CYP6B* genes were differentially regulated by plant allelochemicals [9]. In this observation, we reported a third set of paralogous *CYP321A2*/*CYP321A1*, which are arranged in a tandem cluster in the *H. zea* genome. As documented here, these two *CYP321A* genes share several commonalities in their structure and their allelochemical-induced expression patterns. Both *CYP321A2* and *CYP321A1* lack introns, code for a P450 of 498–499 amino acids, and share relatively high amino acid sequence identity. Both of them were constitutively expressed at a very low level in midgut and fat body, but significantly induced by an array of plant allelochemicals and plant signaling molecules, among which flavone and coumarin caused the highest induction for both genes. Considering their high level of amino acid identity, conserved genomic organization and similar induction features by plant toxins, we speculate that both *CYP321A* genes originated from a common ancestor P450 gene through gene duplication and two *CYP321A* paralogs potentially function as a xenobiotic-metabolizing cytochrome P450 gene during evolutionary history. The hypothesis now has in part been validated by several studies demonstrating that *CYP321A1* is responsible for detoxification of plant toxins and insecticides by baculovirus-mediated functional expression analysis [19,23,24]. Further experiments are necessary to test if *CYP321A2* is also involved in the detoxification of plant allelochemicals.

Gene duplication is a very common phenomenon in all eukaryotic organisms that may occur in several different ways [35] and represents an important process for functional innovation during evolution [36]. The presence of a second copy of a gene would develop unique new opportunities in evolution by allowing one of the two duplicate gene copies to evolve new functional properties [36]. The *CYP321A2*/*CYP321A1* paralogs described here are appropriate for the situation. Baculovirus-mediated expression of the full-length *CYP321A1* cDNA has demonstrated that CYP321A1 protein is able to metabolize its two significant inducers xanthotoxin and flavone [19]. Although the inducibility of P450 is not necessarily correlated to detoxification capability [37], a lot of xenobiotics which have capability to induce the expressions of P450s have shown to be metabolized by corresponding P450s (see Feyereisen, 2012, for a review) [6]. The fact that *CYP321A2* can also be induced by chlorogenic acid, indole-3-carbinol, rutin, quercetin and coumarin in addition to xanthotoxin and flavone implies that CYP321A2 could have the potential to metabolize these allelochemicals. A variety of site-directed mutagenesis studies of closely related P450 proteins have suggested that hypervariable sequences within six substrate recognition sites (SRS) account for the variations in the substrate specificities of P450 proteins [38]. Amino acid sequence comparison showed that all the six SRSs are divergent between *CYP321A2* and *CYP321A1* (Figure 2B), arguing for that *CYP321A2* and *CYP321A1* could have different substrate specificities. Furthermore, divergence in the induced expression patterns of the *CYP321A1/CYP321A2* paralogs potentially assists in the acquisition of different functions for these two genes. The fact that expression pattern divergence is consistent with the divergence of coding sequence between the paralogs indicates that expression pattern coevolved with coding sequence although they may evolve at different rate. Duplicate genes can be preserved by natural selection for gene dosage, thus allowing an increased production of the ancestral gene product [36]. Under stressful environments, the wild-type intact *CYP321A2* may be selected against naturally occurring plant allelochemicals and synthetic insecticides. Future heterologous expression of *CYP321A2* will allow testing if it evolves new functional properties compared to its paralog *CYP321A1*.

It is reported that chlorogenic acid mediate the synthesis of a wide range of phenylpropanoid allelochemicals that is elicited by wounding and that indole-3-carbinol does not normally accumulate in large quantities in intact plant tissue until the tissue is damaged [39]. The results here showed that both *CYP321A* paralogs were significantly induced by chlorogenic acid and indole-3-carbinol (Table 1). Over-expression of these two genes responding to plant substances produced by tissue damage may well pre-activate insect defense systems in response to induced toxin synthesis in the host plants. By responding to plant signaling substances as well as the end-product allelochemicals, insects are able to equip themselves before (or concomitant with) the accumulation of toxic concentrations of plant defense compounds [16]. The *CYP6B* family members (*CYP6B8*, *CYP6B9*, *CYP6B27* and *CYP6B28*) of *H. zea* can be induced by plant-produced signals jasmonate and salicylate in both fat body and midgut [16]. Similarly, our data indicated that jasmonate and salicylate effectively induced the expression of *CYP321A1* in fat body and *CYP321A2* in midgut, respectively (Table 1), which allow insect to “eavesdrop” on plant defense signals to protect *H. zea* against plant toxins. Induction of these broad-substrate enzymes by plant signal molecules allow this generalist to maximize its capacity to detoxify allelochemicals in advance of their biosynthesis by its various host plants.

Different from *CYP321A1*, the array of induced responses of *CYP321A2* indicate that its expression is not induced exclusively by the plant allelochemicals most commonly encountered by this polyphagous feeder. Xanthotoxin, a linear furanocoumarin which is rarely encountered by *H. zea* [40], is a strong inducer of the *CYP321A2* transcript (Table 1). In contrast, some widely distributed allelochemicals, like the glycoside rutin, the aglycone quercetin, and an occasionally encountered glucobrassicin breakdown product present in the Brassicaceae, indole-3-carbinol, are relatively weak inducers of *CYP321A2* transcripts in comparison with xanthotoxin, although the widely distributed flavone and coumarin are the most significant inducers (Table 1). In addition, despite being present in a preferred host plant family for *H. zea*, the sesquiterpene gossypol, which is restricted in distribution to the Malvaceae, is not an effective inducer not only for *CYP321A2* but also for *CYP321A1* (Table 1).

The broad and variable reactions of *CYP321A2* to allelochemicals indicate that the projected ecological encounter rate is just one factor determining the induction efficiency of a particular allelochemical. Other key factors that can determine the induction efficiency of these natural xenobiotics are the numbers of signal cascades and promoter elements that mediate induction of the two P450s by these allelochemicals [9]. This notion can interpret, at least in part, the qualitative and quantitative difference in their induction patterns. For example, only one of the two *CYP321A* genes was induced by 2-tridecanone, jasmonate and salicylate. The two *CYP321A* genes also differed significantly in their induction folds by several allelochemicals, especially xanthotoxin, indole-3-carbinol and quercetin. This observation suggests that multiple receptors and signaling cascades may differentially activate their expressions in response to these diverse inducers and that the structural complexity of allelochemicals may represent another important factor determining induction efficiency.

Once P450s that are inducible by allelochemicals are identified along with their corresponding inducers, the next step is to search for specific *cis*-acting elements for expression regulation in the 5′ flanking region of these genes. XRE-Fla, the essential element mediating flavone- and xanthotoxin-induction of *H. zea CYP321A1* expression, consists of three motifs (the TAAT inverted repeat, the GCT mirror repeat and the ARE-like element) [31,32]. Consistent with the greater induction folds of *CYP321A2* than *CYP321A1* (Table 1) by flavone and xanthotoxin, three copies of XRE-Fla-like element are found in the 5′ promoter region of *CYP321A2* (Figure 5). The first copy (A copy in Figure 5) has exactly the same arrangement of the three component motifs as in the XRE-Fla of *CYP321A1*, strongly implying that this copy should be responsible at least partly for stronger induction of *CYP321A2* by flavone and xanthotoxin. The other two copies exchange the positions of the inverted repeat and the mirror repeat (B and C copies in Figure 5). How such an inversion of the first two repeat motifs affect the induction activity of the B and C copies needs further experimental verification. Nonetheless, the fact that the flavone and xanthotoxin induction folds of *CYP321A2* in midguts and fat bodies were 1.6–13 times those of *CYP321A1* (Table 1) implies that all the three copies of XRE-Fla-like element are functional for the up-regulation of *CYP321A2*.

Overall, this study describes the identification and features of *CYP321A2* as well as the responses of *CYP321A2* and its paralog *CYP321A1* to allelochemicals and plant signal molecules in *H. zea,* a polyphagous pest species. The variations in the expression observed in *CYP321A* genes with similar sequence provide insights into the relative importance of the acquisition of novel detoxificative enzymes and/or novel regulatory pathways in allowing herbivorous insects to colonize new host plants. Further studies on the function of the *CYP321A2* and its regulatory mechanism are required to better understand its role in facilitating *H. zea* to colonize a wide range of host plants.

## 5. Conclusions

The present study reports on the identification of the wild-type intact allele of *CYP321A2* from *H. zea*. This *CYP321A2* allele encodes a deduced protein of 498 amino acids and has the P450 signature motifs. This *CYP321A2* was highly expressed in midgut and fat body and achieved the highest expression levels in the developmental stages of 5th and 3rd instar larvae. *CYP321A2* and *CYP321A1* were constitutively expressed in low levels but can be differentially and significantly induced by a range of plant allelochemicals and plant signaling molecules, among which xanthotoxin, flavone and coumarin were the most prominent inducers of *CYP321A2* both in midguts and fat bodies, whereas flavone, coumarin and indole-3-carbinol were the prominent inducers of *CYP321A1* in midguts and fat bodies. Moreover, the xanthotoxin- and flavone-responsive regulatory element XRE-Fla of *CYP321A1* was also detected in the promoter region of *CYP321A2*. Our results enrich the P450 inventory by identifying an allelochemical broadly induced *CYP321A2*, a paralog of *CYP321A1* in *H. zea*. Our data also suggest that the *CYP321A2*/*CYP321A1* paralogs are a pair of duplicated genes of multigene families and *CYP321A2* could potentially be involved in the detoxification of plant allelochemicals and adaptation of *H. zea* to its chemical environment.

## Figures and Tables

**Table 1:** Induction folds of *CYP321A2* and *CYP321A1* in midgut and fat body by different allelochemicals and plant signal molecules. Thirty newly molted 5th instar larvae were allowed to feed on control diet or control diet containing 0.1% allelochemicals (xanthotoxin, chlorogenic acid, indole-3-carbinol, flavone, rutin, gossypol, 2-tridecanone, quercetin and coumarin) or plant signal molecules (jasmonate and salicylate) for 48 h. The mRNA level was normalized using *EF-1α* as a reference gene. The induction folds of *CYP321A1* and *CYP321A2* shown in this table are obtained by division of the normalized expression levels of the two genes in the treatment groups (larvae fed on control diet containing 0.1% allelochemicals or plant signal molecules) by that of the two genes in the control group (larvae fed on control diet). The numbers show the mean and standard error of the induction fold based on three biological replicates. Induction fold values highlighted in light grey are significantly different from the control group as measured by two-fold accumulation over the level in control group (Student’s *t*-tests, *p* < 0.05).

Types of Plant-Derived Compounds		*CYP321A2*		*CYP321A1*	
		Midgut	Fat Body	Midgut	Fat Body
plant allelochemicals	Xanthotoxin	60.42 ± 14.70	20.62 ± 4.37	4.55 ± 0.74	7.94 ± 2.42
	chlorogenic acid	12.86 ± 8.05	10.58 ± 2.21	7.05 ± 0.87	4.60 ± 0.73
	indole-3-carbinol	7.13 ± 1.64	2.60 ± 0.30	33.74 ± 4.01	16.59 ± 3.17
	Flavone	850.76 ± 389.11	16.99 ± 3.18	306.82 ± 15.38	10.36 ± 1.92
	Rutin	12.58 ± 2.62	1.06 ± 0.37	24.55 ± 1.21	3.54 ± 0.90
	Gossypol	1.19 ± 0.66	1.23 ± 0.25	1.68 ± 0.47	1.37 ± 0.07
	2-tridecanone	18.25 ± 2.64	0.91 ± 0.40	20.34 ± 1.40	2.24 ± 0.28
	Quercetin	11.88 ± 3.69	2.13 ± 1.44	40.44 ± 2.66	4.25 ± 0.25
	Coumarin	61.18 ± 26.38	12.37 ± 2.16	102.36 ± 4.46	6.04 ± 0.99
plant signaling molecules	Jasmonate	3.60 ± 1.34	0.45 ± 0.18	0.81 ± 0.13	2.65 ± 0.08
	Salicylate	2.06 ± 0.20	0.61 ± 0.40	1.80 ± 0.42	1.55 ± 0.11

## Data Availability

There is no additional data to disclose, all data are included in this manuscript.

## References

[1] Berenbaum M.R. (1990). Evolution of specialization in insect-umbellifer associations. Annu. Rev. Entomol..

[2] Bernays E.A., Chapman R.F. (1994). Host-Plant. Selection by Phytophagous Insects.

[3] Gatehouse J.A. (2002). Plant resistance towards insect herbivores: A dynamic interaction. New Phytol..

[4] Li X., Baudry J., Berenbaum M.R., Schuler M.A. (2004). Structural and functional divergence of insect CYP6B proteins: From specialist to generalist cytochrome P450. Proc. Natl. Acad. Sci. USA.

[5] Cohen M.B., Schuler M.A., Berenbaum M.R. (1992). A host-inducible cytochrome P450 from a host-specific caterpillar: Molecular cloning and evolution. Proc. Natl. Acad. Sci. USA.

[6] Feyereisen R., Gilbert L.I. (2012). Insect CYP Genes and P450 Enzymes. Insect Molecular Biology and Biochemistry.

[7] Schuler M.A. (1996). The role of cytochrome P450 monooxygenases in plant-insect interactions. Plant Physiol..

[8] Berenbaum M.R. (2002). Postgenomic chemical ecology: From genetic code to ecological interactions. J. Chem. Ecol..

[9] Li X., Schuler M.A., Berenbaum M.R. (2002). Plant allelochemicals differentially regulate *Helicoverpa zea* cytochrome P450 genes. Insect Mol. Biol..

[10] Hansen T.F., Carter A.J., Chiu C.H. (2000). Gene conversion may aid adaptive peak shifts. J. Theor. Biol..

[11] Teshima K.M., Innan H. (2004). The effect of gene conversion on the divergence between duplicated genes. Genetics.

[12] Osada N., Innan H. (2008). Duplication and gene conversion in the *Drosophila melanogaster* genome. PLoS Genet..

[13] Ohta T. (2010). Gene conversion and evolution of gene families: An overview. Genes.

[14] Walsh J.B. (1987). Sequence-dependent gene conversion: Can duplicated genes diverge fast enough to escape conversion?. Genetics.

[15] Li X., Berenbaum M.R., Schuler M.A. (2002). Cytochrome P450 and actin genes expressed in *Helicoverpa zea* and *Helicoverpa armigera*: Paralogy/orthology identification, gene conversion, and evolution. Insect Biochem. Mol. Biol..

[16] Li X., Schuler M.A., Berenbaum M.R. (2002). Jasmonate and salicylate induce expression of herbivore cytochrome P450 genes. Nature.

[17] Feyereisen R. (1999). Insect P450 enzymes. Annu. Rev. Entomol..

[18] Tijet N., Helvig C., Feyereisen R. (2001). The cytochrome P450 gene superfamily in *Drosophila melanogaster*: Annotation, intron–exon organization and phylogeny. Gene.

[19] Sasabe M., Wen Z., Berenbaum M.R., Schuler M.A. (2004). Molecular analysis of *CYP321A1*, a novel cytochrome P450 involved in metabolism of plant allelochemicals (furanocoumarins) and insecticides (cypermethrin) in *Helicoverpa zea*. Gene.

[20] Zeng R., Wen Z., Niu G., Schuler M.A., Berenbaum M.R. (2007). Allelochemical induction of cytochrome P450 monooxygenases and amelioration of xenobiotic toxicity in *Helicoverpa zea*. J. Chem. Ecol..

[21] Zeng R., Wen Z., Niu G., Schuler M.A., Berenbaum M.R. (2009). Enhanced toxicity and induction of cytochrome P450s suggest a cost of “eavesdropping” in a multitrophic interaction. J. Chem. Ecol..

[22] Wen Z., Zeng R., Niu G., Berenbaum M.R., Schuler M.A. (2009). Ecological significance of induction of broad-substrate cytochrome P450s by natural and synthetic inducers in *Helicoverpa zea*. J. Chem. Ecol..

[23] Rupasinghe S.G., Wen Z., Chiu T., Schuler M.A. (2007). *Helicoverpa zea CYP6B8* and *CYP321A1*: Different molecular solutions to the problem of metabolizing plant toxins and insecticides. Protein Eng. Des. Sel..

[24] Niu G., Wen Z., Rupasinghe S.G., Zeng R., Berenbaum M.R., Schuler M.A. (2008). Aflatoxin B1 detoxification by *CYP321A1* in *Helicoverpa zea*. Arch. Insect Biochem..

[25] Waldbauer G.P., Cohen R.W., Friedman S. (1984). An improved procedure for laboratory rearing of the corn earworm, *Heliothis zea* (Lepidoptera: Noctuidae). Great Lakes Entomol..

[26] Sambrook J., Russell D.W. (2001). Molecular Cloning: A Laboratory Manual.

[27] Sambrook J., Fritsch E.F., Maniatis T. (1989). Molecular Cloning: A Laboratory Manual.

[28] Livak K.J., Schmittgen T.D. (2001). Analysis of relative gene expression data using real-time quantitative PCR and the 2(-delta delta C(T)) method. Methods.

[29] Hasemann C.A., Kurumbail R.G., Boddupalli S.S., Peterson J.A., Deisenhofer J. (1995). Structure and function of cytochromes P450: A comparative analysis of three crystal structures. Structure.

[30] Werck-Reichhart D., Feyereisen R. (2000). Cytochromes P450: A success story. Genome Biol..

[31] Zhang C., Luo X., Ni X., Zhang Y., Li X. (2010). Functional characterization of *cis*-acting elements mediating flavone-inducible expression of *CYP321A1*. Insect Biochem. Mol. Biol..

[32] Zhang C., Wong A., Zhang Y., Ni X., Li X. (2014). Common and unique *cis*-acting elements mediate xanthotoxin and flavone induction of the generalist P450 *CYP321A1*. Sci. Rep..

[33] Li X., Berenbaum M.R., Schuler M.A. (2007). Molecular mechanisms of metabolic resistance to synthetic and natural xenobiotics. Annu. Rev. Entomol..

[34] Hodgson E., Kerkut G.A., Gilbert L.I. (1985). Microsomal Monooxygenases. Comprehensive Insect Physiology, Biochemistry and Pharmacology.

[35] Lynch M. (2007). The Origins of Genome Architecture.

[36] Ohno S. (1970). Evolution by Gene Duplication.

[37] Wang H., Shi Y., Wang L., Liu S., Wu S., Yang Y., Feyereisen R., Wu Y. (2018). *CYP6AE* gene cluster knockout in *Helicoverpa armigera* reveals role in detoxification of phytochemicals and insecticides. Nat. Commun..

[38] Gotoh O. (1992). Substrate recognition sites in cytochrome P450 family 2 (CYP2) proteins inferred from comparative analyses of amino acid and coding nucleotide sequences. J. Biol. Chem..

[39] Seigler D.S. (1998). Plant Secondary Metabolism.

[40] Kogan J.D., Sell K., Stinner R.E., Bradley J.R., Kogan M. (1978). A Bibliography of Heliothis zea (Boddie) and H. virescens (F.) (Lepidoptera: Noctuidae).

